# Genome-wide investigation to assess copy number variants in the Italian local chicken population

**DOI:** 10.1186/s40104-023-00965-7

**Published:** 2024-01-03

**Authors:** Filippo Cendron, Martino Cassandro, Mauro Penasa

**Affiliations:** 1 Department of Agronomy, Food, Natural Resources, Animals and Environment (DAFNAE), University of Padova, Viale Dell’Università 16, 35020 Legnaro, PD Italy; 2 Federazione Delle Associazioni Nazionali Di Razza E Specie, Via XXIV Maggio 43, 00187 Rome, Italy

**Keywords:** Chicken, Copy number variant, Conservation, Local breed, SNP

## Abstract

**Background:**

Copy number variants (CNV) hold significant functional and evolutionary importance. Numerous ongoing CNV studies aim to elucidate the etiology of human diseases and gain insights into the population structure of livestock. High-density chips have enabled the detection of CNV with increased resolution, leading to the identification of even small CNV. This study aimed to identify CNV in local Italian chicken breeds and investigate their distribution across the genome.

**Results:**

Copy number variants were mainly distributed across the first six chromosomes and primarily associated with loss type CNV. The majority of CNV in the investigated breeds were of types 0 and 1, and the minimum length of CNV was significantly larger than that reported in previous studies. Interestingly, a high proportion of the length of chromosome 16 was covered by copy number variation regions (CNVR), with the major histocompatibility complex being the likely cause. Among the genes identified within CNVR, only those present in at least five animals across breeds (*n* = 95) were discussed to reduce the focus on redundant CNV. Some of these genes have been associated to functional traits in chickens. Notably, several CNVR on different chromosomes harbor genes related to muscle development, tissue-specific biological processes, heat stress resistance, and immune response. Quantitative trait loci (QTL) were also analyzed to investigate potential overlapping with the identified CNVR: 54 out of the 95 gene-containing regions overlapped with 428 QTL associated to body weight and size, carcass characteristics, egg production, egg components, fat deposition, and feed intake.

**Conclusions:**

The genomic phenomena reported in this study that can cause changes in the distribution of CNV within the genome over time and the comparison of these differences in CNVR of the local chicken breeds could help in preserving these genetic resources.

**Supplementary Information:**

The online version contains supplementary material available at 10.1186/s40104-023-00965-7.

## Background

Copy number variants (CNV) are structural genomic alterations distributed across the entire genome in all species, with a mean size of at least 50 bp [1, 2], and they are caused by insertions, deletions, duplications, and translocations of DNA fragments [2, 3]. The opportunity to sequence whole genomes has facilitated the use of molecular markers to characterize the breeds; indeed, structural variations of the genome have an important role in gene expression and genome evolution within populations. As with microsatellites and single nucleotide polymorphisms (SNP), CNV can be used to investigate genetic variation and diversity [4–6]. In all species, CNV can intersect genes, altering their structure and expression, and causing phenotypic variability and disease susceptibility in humans [7, 8] and animals [6, 9, 10]. The CNV can explain a large portion of the loss of heritability in genome-wide studies for some traits [11, 12]. Although CNV are less prevalent in the genome than other molecular markers, they cover a larger portion of the genome and thus can have powerful effects on phenotypic variability [13, 14]. In contrast to SNP, CNV can span larger genomic regions and have greater mutation rate, potentially exerting substantially more influence on gene structure, regulation, and expression [15].

Several studies in chickens have pinpointed quantitative trait loci (QTL) and positional candidate genes marked by significantly associated SNP for economically important traits, including growth performance, carcass characteristics, and abdominal fat deposition [15]. It is unsurprising that the number of studies dealing with CNV has also increased within chicken populations [15]. Notably, CNV linked to traits such as late feathering, the pea-comb phenotype, dermal hyperpigmentation, dark brown plumage color, and resistance/susceptibility to Marek's disease have been documented [15].

The CNV has a pivotal role in the evolutionary adaptation of organisms and influence their fitness and reproductive capabilities under both natural and artificial selection pressures. This underscores their significance as a substantial source of adaptive potential. For instance, the copy number of AMY1 demonstrates a robust correlation with dietary evolution, with individuals historically consuming high-starch diets typically having more AMY1 copies than those consuming low-starch diets [16]. Furthermore, Minias et al. [17] have reported that the evolution of major histocompatibility complex (MHC) copy number in birds is driven by selective pressures, including those arising from intra- and extra-cellular pathogens and parasites. Copy number variants have also been implicated in the phenotypic variability of traits crucial to domestication and breed development in livestock species. For instance, the duplication of *KIT* gene is significantly associated with white coat color in both pigs and cattle and the chicken pea-comb phenotype is attributed to the duplication of SOX5 within intron 1 [16].

Copy number variants in the poultry genome have often been mapped using low-density chips [18, 19] or a limited sample size [6]. In recent years, the use of 600 K density chips in the chicken has allowed researchers to obtain a significant number of outputs useful for more accurate detection of CNV [6]. Although whole-genome sequence data can improve the identification of smaller CNV (unlike the SNP-array-based approach), this is economically disadvantageous to be performed at population level [20, 21].

The aims of this study were to investigate i) the type and amount of CNV and CNV regions (CNVR), and ii) the genes that undergo the effect of their presence with an unprecedented resolution using a high-density SNP chip in a large sample of local Italian chickens. Finally, the genetic variability and CNV, CNVR, and genes in common amongst breeds were characterized.

## Methods

### Sampling and genotyping

The DNA from 508 individuals from 23 local Italian chicken breeds (approximately 22 individuals per breed; Table 1) were genotyped using the Affymetrix Axiom 600 K Chicken Genotyping Array (for full details see Cendron et al. [5]). Local breeds were Ancona (ANC), Bianca di Saluzzo (BSA), Bionda Piemontese (BPT), Cornuta di Caltanissetta (COR), Livorno Bianca (PLB), Livorno Nera (PLN), Mericanel della Brianza (MER), Modenese (MOD), Mugellese (MUG), Ermellinata di Rovigo (PER), Millefiori di Lonigo (PML), Padovana Argentata (PPA), Polverara Bianca (PPB), Padovana Camosciata (PPC), Padovana Dorata (PPD), Polverara Nera (PPN), Pepoi (PPP), Robusta Lionata (PRL), Robusta Maculata (PRM), Romagnola (ROM), Siciliana (SIC), Valdarnese (VLD), and Valplatani (VLP).

**Table 1:** Descriptive statistics of copy number variants identified in the Italian local chicken breeds

	**Type**									
**Breed**	**0**	**1**	**3**	**4**	**Total**	**Length**	**Mean length**	**Minimum length**	**Maximum length**	**Genome coverage, %**
ANC	24	427	27	2	480	30,895,153	64,365	2,378	918,621	2.71
BPT	27	104	8	0	139	9,930,942	71,446	5,580	1,862,447	0.87
BSA	58	348	11	0	417	44,933,947	107,755	6,464	803,891	3.94
COR	31	196	43	0	270	21,300,170	78,890	857	923,575	1.87
MER	32	124	4	0	160	15,414,093	96,338	5,157	900,960	1.35
MOD	15	54	9	0	78	2,324,960	29,807	4,494	100,019	0.20
MUG	46	150	15	0	211	10,431,587	49,439	2,726	506,421	0.91
PER	90	22	7	0	119	5,647,643	51,813	7,526	472,494	0.49
PLB	36	96	9	0	141	8,284,013	58,752	4,389	304,806	0.73
PLN	38	152	5	0	195	10,091,542	51,752	1,643	347,761	0.88
PML	134	198	13	0	345	24,815,055	71,928	4,431	412,846	2.17
PPA	15	39	8	0	62	3,095,619	49,929	9,035	153,514	0.27
PPB	24	31	8	1	64	1,847,570	28,868	3,023	115,628	0.16
PPC	34	39	5	0	78	2,284,986	33,603	3,023	221,819	0.20
PPD	27	35	18	0	80	2,276,698	28,459	3,023	221,819	0.20
PPN	23	26	2	0	51	527,861	12,875	3,023	45,506	0.05
PPP	91	344	0	0	435	75,993,667	174,698	4,904	2,863,848	6.66
PRL	9	25	6	0	40	941,152	23,529	3,614	68,519	0.08
PRM	40	30	17	1	88	2,075,529	27,310	2,378	69,072	0.18
ROM	6	74	16	8	104	4,068,436	39,120	6,264	217,674	0.36
SIC	65	104	15	0	184	8,853,192	48,115	5,105	256,437	0.78
VLD	13	33	7	0	53	4,929,325	94,795	3,499	1,059,613	0.43
VLP	52	409	7	0	468	79,647,292	170,187	3,249	2,849,628	6.98
Total^*^	930	3,060	260	12	4,262	370,610,432	1,463,770	95,785	15,696,918	32

^*^Common and not common copy number variants

*ANC* Ancona, *BSA* Bianca di Saluzzo, *BPT* Bionda Piemontese, *COR* Cornuta di Caltanissetta, *PLB* Livorno Bianca, *PLN* Livorno Nera, *MER* Mericanel della Brianza, *MOD* Modenese, *MUG* Mugellese, *PER* Ermellinata di Rovigo, *PML* Millefiori di Lonigo, *PPA* Padovana Argentata, *PPB* Polverara Bianca, *PPC* Padovana Camosciata, *PPD* Padovana Dorata, *PPN* Polverara Nera, *PPP* Pepoi, *PRL* Robusta Lionata, *PRM* Robusta Maculata, *ROM* Romagnola, *SIC* Siciliana, *VLD* Valdarnese, *VLP* Valplatani

Before running the CNV calling, the raw genotype dataset underwent a quality check using the Axiom Analysis Suite Software (Affymetrix) to remove the SNP with call rate < 97% and Dish Quality Control < 82%. The final dataset contained 508 animals and 472,821 SNP targets.

### Identification of CNV

The Axiom CNV summary software tool was used to create input files for CNV calling in PennCNV software, which utilizes the Log R ratio (LRR) and B allele frequency (BAF) [22–24]. Prior to PennCNV calling, the raw CNV were visualized using the Axiom CNV Viewer software. The individual-based CNV calling was then carried out using the default parameters of the Hidden Markov Model, i.e., a standard deviation of LRR < 0.30, BAF drift set to 0.01, waviness factor at 0.05, and minimum of 3 SNP to define a CNV. The distribution of CNV per individual spanned from 0 to > 100 [6]. To avoid the detection of false positive and/or negative CNV and outliers, different “.hmm” files (agre.hmm, affygw6.hmm, hh550.hmm) were used to run PennCNV as described by Strillacci et al. [2], Gorla et al. [6], and Fernandes et al. [15]. Indeed, the “.hmm” file may substantially affect the false positive and the false negative rate. The PennCNV manual (https://penncnv.openbioinformatics.org/en/latest/) declares that the agre.hmm file returns more false positive calls than the affygw6.hmm file, which produces a lower number of CNV calls. The analysis was conducted also using the hh550.hmm file as the default method for the calling [2]. To obtain a valid CNV, the common calls from all the three hidden Markov models were considered [6, 15]. This solved the critical choice of which.hmm output file is more appropriate to map CNV to control false positive and negative calls (Additional file 1). Genomic waves were adjusted using the chicken GC model file, which was generated by calculating the GC content of 1-Mb genomic regions surrounding each marker (500 kb on each side), after the program argument ‘gcmodel’ was used to adjust the results [25]. In addition, to validate the outputs from PennCNV, the optimal segmenting module of SVS 8.7.0 (Golden Helix Inc., Bozeman, MT, USA) was used to identify CNV through the univariate approach that segments each sample independently. Quality assurance of the LRR data and filtering of outlier samples were performed using SVS software following the approach of Pinto et al. [26]. Individuals were screened for their GC content, which is correlated to long-range waviness of LRR. Outlying samples were detected by the SVS 8.7.0 for waviness and those identified were deleted [26]. The CNV identified through the two algorithms were merged and consensus among the outputs were used to identify the final CNV for further analysis (Additional file 1).

### Summary of CNV and definition of CNVR

The R package HandyCNV [27] was used on PennCNV output files to summarize CNV and define CNVR. The following package commands were imputed to the analysis: i) *cnv_clean ()* function to convert CNV results into a standard format and make basic summary (the CNV larger than 5 Mb were discarded) [28]; ii) *cnv_summarise_plot ()* to create the CNV distribution, frequency, and length group plot; iii) *call_cnvr ()* to define the CNVR and their frequency (merging CNV that overlapped by at least 1 bp) [28]. In the CNVR map and definition, “gain” indicates the regions that contain more than two copies of CNV, “loss” indicates the regions that contain deleted CNV, and “mixed” the regions that contain at least one duplicated and one deleted CNV. Consensus CNVR were generated with the *call_cnvr ()* command by combining the identified CNVR and the overlapping regions in the final CNVR distribution map as described in Zhou et al. [28].

### Chromosomal distribution and annotation of CNVR

The CNVR distribution map was created through the command *cnvr_plot ()* in HandyCNV R package [28]. Both CNV and CNVR were annotated using *get_refgene ()* and *call_gene ()* functions*,* to obtain reference genome and genes, respectively, based on formatted reference gene list of *Gallus gallus 6.0* chicken assembly (UCSC Genome Browser GRCg6a—https://tinyurl.com/2unb8sf3).

The gene frequency was estimated during the annotation process through of counting the total number of CNV that were annotated to intersect the gene. At the end, the genes under CNV presence were only considered if they were observed in more than five individuals between the breeds [29]. The Gene Ontology (GO) and QTL were identified using Panther algorithm (http://www.pantherdb.org/) and Animal QTLdb database (https://www.animalgenome.org/cgi-bin/QTLdb/index), respectively. The Database for Annotation, Visualization, and Integrated Discovery (DAVID, version 6.8; https://david.ncifcrf.gov/) was used to perform the GO enrichment analysis and the Kyoto Encyclopedia of Genes and Genomes (KEGG) pathway analysis. The visual plots and figures were obtained through *ggplot2* and *tidyverse* R package [30–32].

### Clustering analysis using CNVR

A clustering analysis using the detected CNVR was performed using the method described in Gorla et al. [6]. The scoring matrix of the CNVR was constructed, giving the “0” or “1” values to identify the presence or absence of CNV, respectively, within a specific CNVR. A hierarchical agglomerative clustering was applied to the scoring matrix using *pvclust* R package [33] and multiscale bootstrap re-sampling (100,000 bootstraps) was used to obtain the approximately unbiased* P*-value (AU-P) and estimate a bootstrap probability *P*-value (BP-P) to determinate the branches’ robustness. The unweighted pair group method with arithmetic mean (UPGMA) was chosen as agglomerative method.

## Results

### Identification of CNV and CNVR

A total of 4,262 common CNV remained after merging results from PennCNV and SVS softwares (Table 1, Additional file 1): 3,990 CNV were deletions (i.e., loss state) and 272 duplications (i.e., gain state).

The ANC, VLP, PPP, and BSA local breeds had the greatest number of CNV, namely 480, 468, 435, and 417, respectively. It is worth reporting that the COR breed had the greatest number of duplicated CNV (43) and ANC the greatest number of CNV losses (451). Total genome coverage by entire CNV presence was greatest in BSA (3.94%), PPP (6.66%), and VLP (6.98%) when considering the 28 autosomes in the GRCg6a. Total genome coverage by entire CNV detected was 32%, with average length of 1,463,770 kb.

**Fig. 1:**
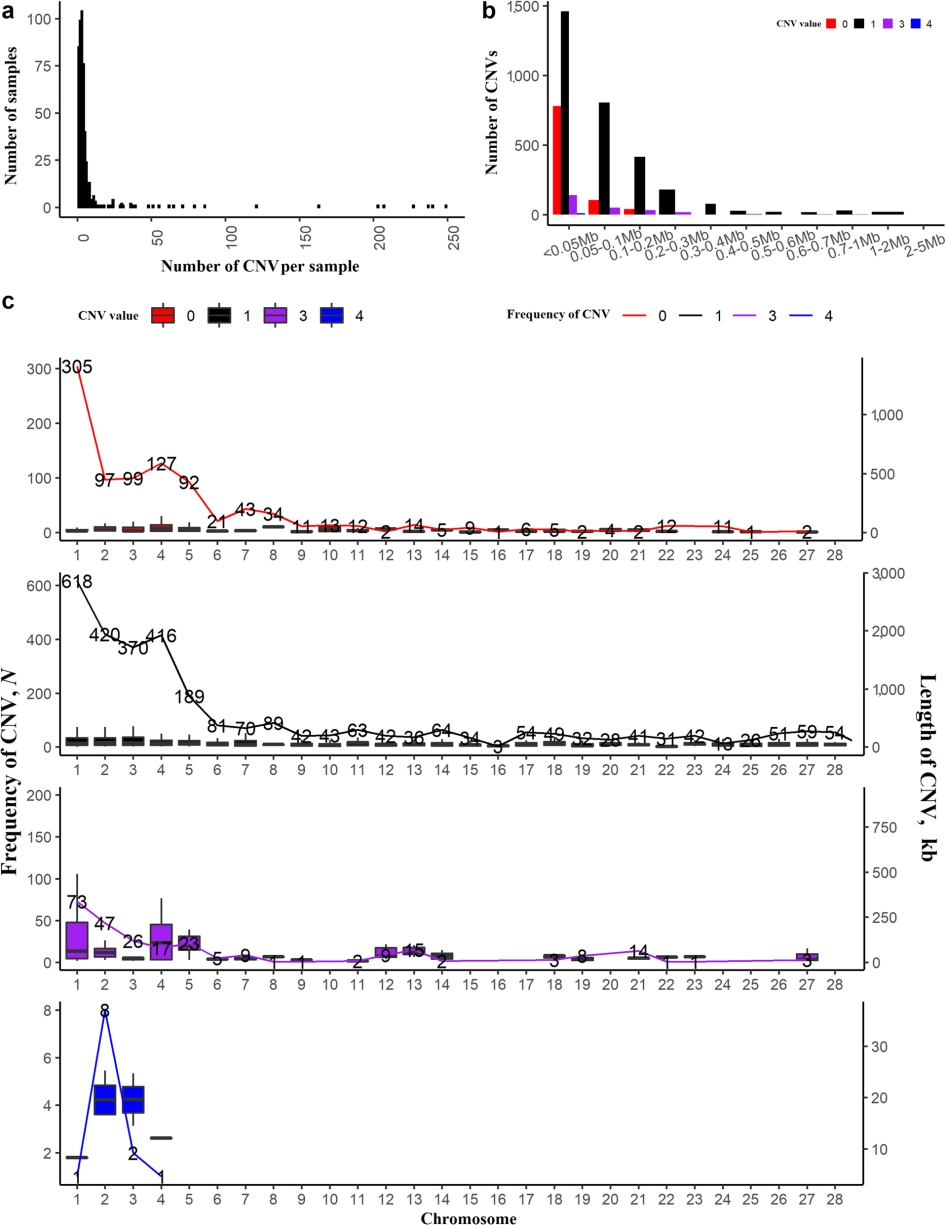
CNV summary plot. **a** Representative result of number of CNV per sample. **b** Frequency of copies of CNV in different length groups. **c** Total CNV state and distributions on chromosomes. The lines indicate the number of CNV and bar plot the length distribution

A summary of the detected CNV is depicted in Fig. 1. Results are presented as distribution of the number of CNV per sample (Fig. 1a), average length of each type of CNV (Fig. 1b), and location on chromosomes (Fig. 1c). The majority of CNV were deletions (0 or 1) with a mean length between 0.05 and 0.1 Mb (Fig. 1a and b). As expected, a high number of CNV were located on the first six autosomes, as they are the largest of the entire genome; noteworthy, no duplicated CNV were detected on chromosomes 10, 15, 16, 17, 24, 25, 26, or 28 (Fig. 1c).

**Fig. 2:**
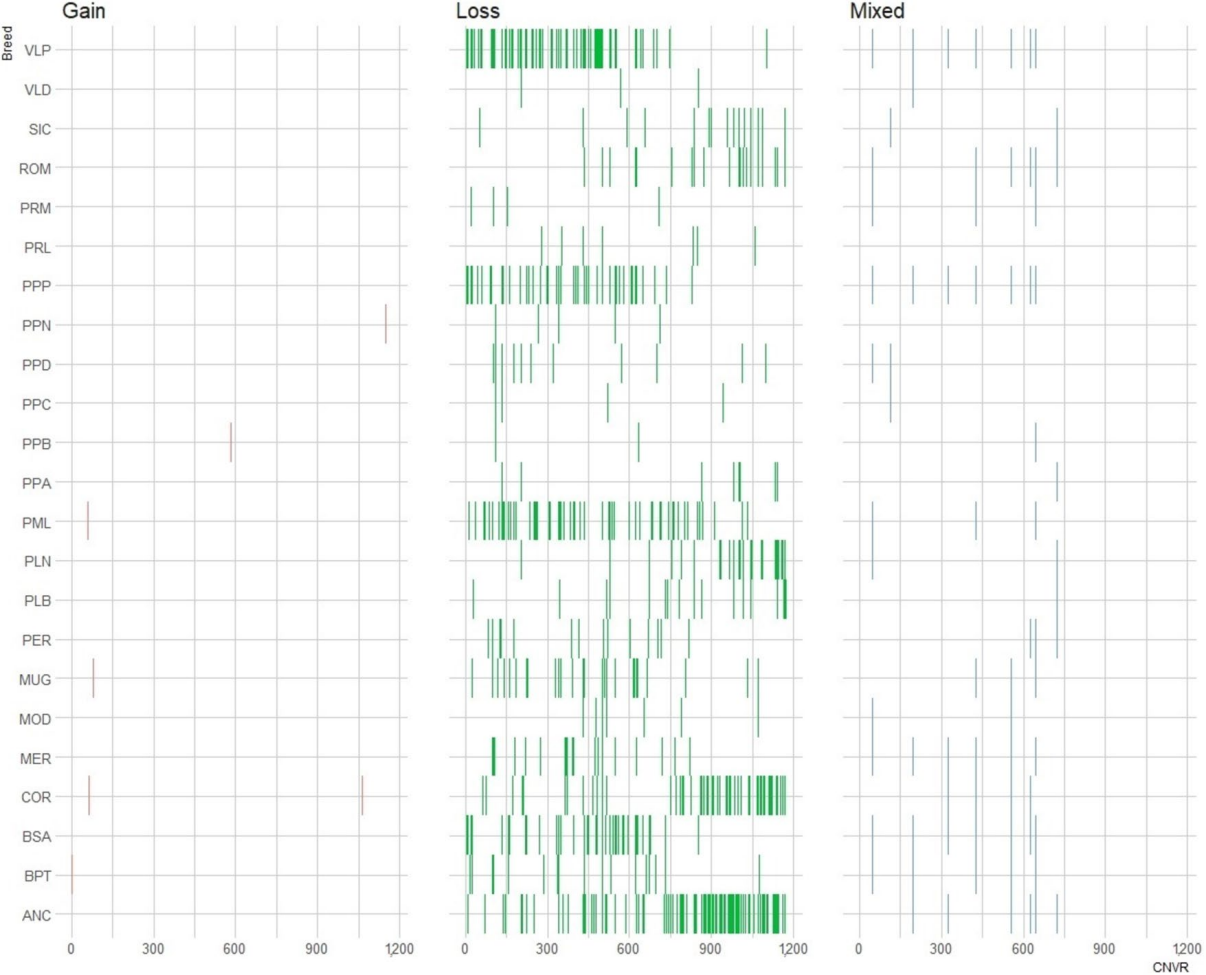
Graphical representation of copy number variation regions among breeds, divided in class of state (purple = gain, green = loss, blue = mixed). Copy number variation regions in progressive number from 1 to 1,172 are represented by lines

The number of identified CNVR by breed is presented in Table 2, along with their state and some quantitative characteristics. These summarized CNV enabled the detection of 1,172 CNVR across all the breeds. These CNVR are comprised of 1,082 losses, 36 gains, and 54 mixed effects. This resulted in 482 common interbreed CNVR and 690 CNVR that were defined as unique to particular breeds (Fig. 2). A high number of loss type CNVR were detected in ANC (345), COR (202), PML (217), and VLP (213), and a high number of gain type CNVR were identified in ANC (7), COR (6), MUG (6), and PPD (7). The CNVR of mixed type were identified in all breeds with greater number in BSA (40), COR (37), and PPP (36). The longest portion of genome covered by a single CNVR was observed in PPP (50,708,742 kb, 4.44% genome coverage), followed by VLP (2.71%), and ANC (2.06%; Table 2). Total genome coverage of identified CNVR was 13.64%, considering 28 autosomes in the GRCg6a.

**Table 2:** Descriptive statistics of copy number variation regions identified in the Italian chicken breeds

	Type								
Breed	Loss	Gain	Mixed	Total	Length	Mean	Min length	Max length	Genome coverage, %
ANC	345	7	28	380	23,529,525	61,920	2,378	565,429	2.06
BPT	54	4	22	80	5,317,732	66,472	5,580	1,862,447	0.47
BSA	137	3	40	180	13,727,398	76,263	6,464	391,642	1.20
COR	202	6	37	245	13,959,094	56,976	8,570	582,168	1.22
MER	84	0	28	112	9,360,221	83,573	5,157	814,780	0.82
MOD	20	3	4	27	648,977	24,036	4,494	77,226	0.06
MUG	99	6	26	131	6,214,065	47,436	2,726	246,916	0.54
PER	54	0	14	68	4,847,736	71,290	7,526	472,494	0.42
PLB	51	2	9	62	3,880,517	62,589	4,389	276,030	0.34
PLN	82	0	10	92	5,010,687	54,464	1,750	347,761	0.44
PML	217	5	22	244	17,840,778	73,118	4,431	333,470	1.56
PPA	30	2	6	38	2,408,448	63,380	9,035	153,514	0.21
PPB	19	6	5	30	757,175	25,239	3,023	110,042	0.07
PPC	19	3	5	27	1,343,003	49,741	3,023	221,819	0.12
PPD	39	7	8	54	1,796,233	33,264	3,023	221,819	0.16
PPN	9	2	1	12	192,241	16,020	3,023	45,506	0.02
PPP	167	0	36	203	50,708,742	249,797	4,904	2,863,848	4.44
PRL	27	2	2	31	689,461	22,241	3,614	66,137	0.06
PRM	18	3	6	27	692,116	25,634	2,378	69,072	0.06
ROM	41	4	5	50	3,862,446	44,396	6,264	160,389	0.34
SIC	40	1	8	49	2,430,370	49,599	5,105	256,437	0.21
VLD	16	3	3	22	1,488,845	67,675	3,499	1,059,613	0.13
VLP	213	5	45	263	30,904,092	117,506	3,249	1,148,469	2.71
Total identify^*^	1,084	36	54	1,172	155,598,588	317,459	1,750	2,929,354	13.64

^*^Number of unique copy number variation regions identified

*ANC* Ancona, *BSA* Bianca di Saluzzo, *BPT* Bionda Piemontese, *COR* Cornuta di Caltanissetta, *PLB* Livorno Bianca, *PLN* Livorno Nera, *MER* Mericanel della Brianza, *MOD* Modenese, *MUG* Mugellese, *PER* Ermellinata di Rovigo, *PML* Millefiori di Lonigo, *PPA* Padovana Argentata, *PPB* Polverara Bianca, *PPC* Padovana Camosciata, *PPD* Padovana Dorata, *PPN* Polverara Nera, *PPP* Pepoi, *PRL* Robusta Lionata, *PRM* Robusta Maculata, *ROM* Romagnola, *SIC* Siciliana, *VLD* Valdarnese, *VLP* Valplatani

Due to the high level of CNVR associated to the loss type, overlapping regions between breeds were observed. Likewise, in the cluster of the mixed type, some overlapping regions were detected. In terms of density, the ANC, PML, PPP, and VLP exhibited the greatest number of loss type CNVR (Fig. 2). Unique CNVR were classified as singleton if detected in only one individual (Table 3). Among the identified CNVR, 690 (58.9%) were singleton, 235 (20.1%) were identified in two individuals, 72 (6.1%) in three individuals, 56 (4.8%) in four individuals, 32 (2.7%) in five individuals, and 87 (7.4%) in six or more individuals.

**Table 3:** Genome covered (%) by copy number variation regions for each chromosome (Chr)

Chr	Loss	Gain	Mixed	Total	Length	Coverage, %^*^
1	180	9	18	207	71,613,929	22.0
2	133	2	10	145	54,589,668	20.3
3	107	2	7	116	40,625,275	17.5
4	111	2	5	118	33,318,169	24.5
5	65	3	3	71	21,836,235	15.9
6	40	1	1	42	12,945,461	9.5
7	36	3	3	42	13,485,632	9.2
8	35	1	0	36	10,936,500	8.1
9	29	1	0	30	8,793,422	5.9
10	30	0	0	30	7,458,900	5.0
11	27	0	1	28	7,379,859	10.3
12	19	1	1	21	7,281,076	7.2
13	19	2	2	23	6,718,723	8.8
14	28	2	0	30	5,692,194	12.8
15	20	0	0	20	4,658,439	7.1
16	4	0	0	4	238,103	36.5
17	20	0	0	20	3,999,086	17.5
18	20	2	0	22	4,034,610	13.6
19	19	2	0	21	3,642,637	11.7
20	21	0	0	21	5,149,920	8.2
21	14	1	0	15	2,504,894	8.1
22	9	1	0	10	1,726,356	16.5
23	15	0	1	16	2,112,083	20.8
24	10	0	0	10	2,292,400	9.3
25	13	0	0	13	1,060,800	27.0
26	22	0	0	22	1,939,526	28.6
27	15	1	2	18	2,064,365	30.6
28	21	0	0	21	1,815,610	28.4
Total	1,082	36	54	1,172		

^*^Coverage of copy number variation regions by chromosome relatively to each chromosome length

**Fig. 3:**
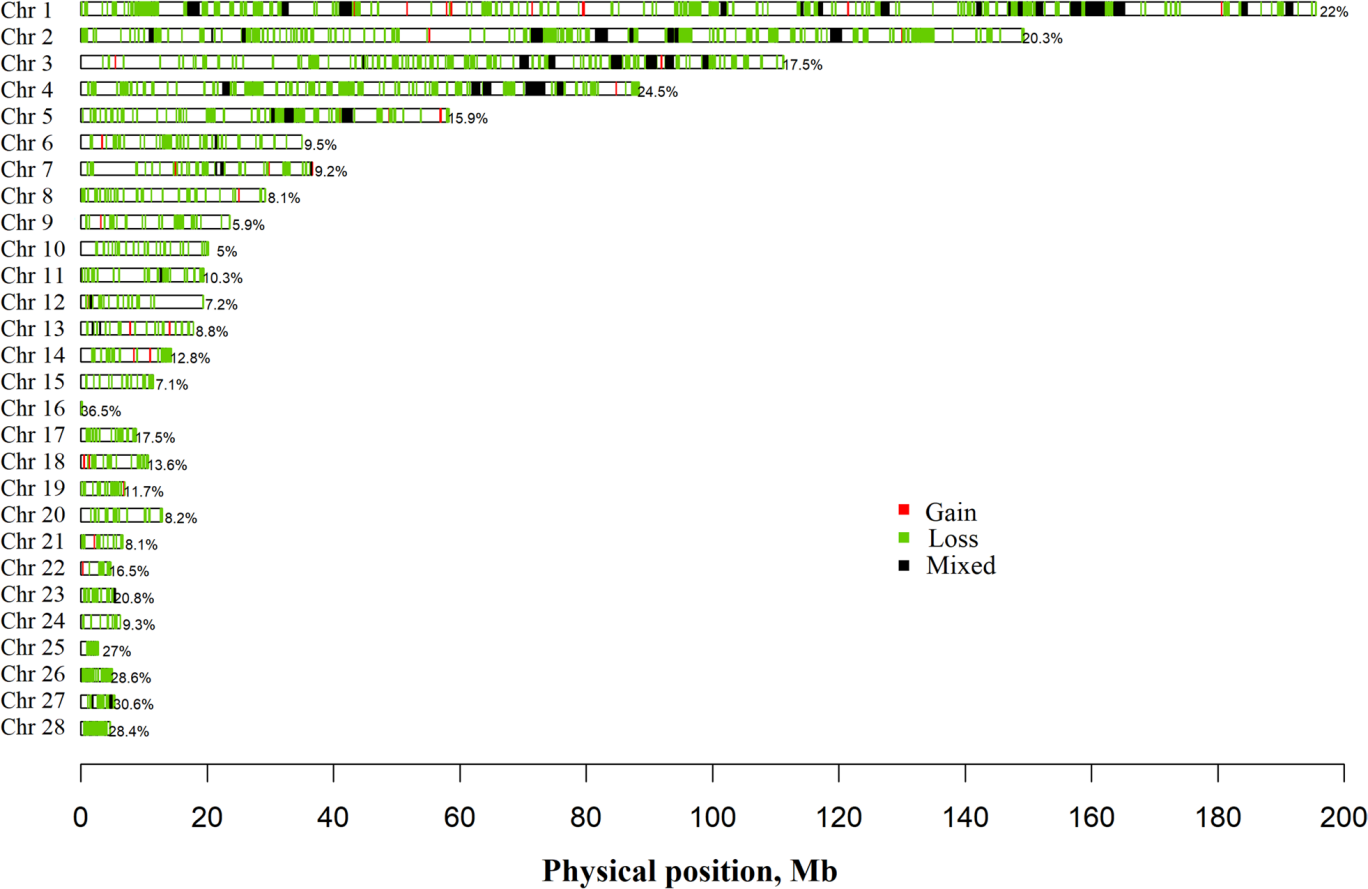
Physical distribution of copy number variation regions on chromosomes, according to state (gain, loss, and mixed)

Figure 3 depicts the CNVR map according to each type (gain, loss, and mixed) on each chromosome. A detailed overview of the distribution of CNVR on chromosomes of the studied breeds is reported in the Additional file 2. The greatest number of CNVR was identified on chromosome 1 and the breeds ANC, PML, PPP, and VLP had the greatest representation. On second, third, and fourth chromosomes, the aforementioned breeds had the greatest number of CNVR, however, the presence of CNVR decreased drastically on the other chromosomes and ANC and COR had the greatest number of CNVR, with ANC being the only breed with a CNVR on chromosome 16 (Additional file 2).

The number of CNVR detected together with the state and the proportion of coverage by chromosomes is reported in Table 3. The proportion of coverage ranged from 5% to 36.5%, with the highest value observed on chromosome 16, which was expected since it is the shortest chromosome of the chicken genome.

The CNVR were grouped according to their length into 4 classes: 1 to 10 kb (*n* = 83), 11 to 100 kb (*n* = 760), 101 to 200 kb (*n* = 155), and > 200 kb (*n* = 174). The greatest number of loss type CNVR were those with length from 11 to 100 kb, and the greatest number of gains and mixed type CNVR were those with length from 11 to 100 kb and > 200 kb, respectively (Additional file 3).

The comparison amongst the CNVR observed in the present study and those of other studies [6, 15, 16, 34–37] is summarized in Table 4. Out of 1,172 CNVR, from 149 to 1,160 overlapped with CNVR identified in previous studies on chicken, meaning that a decent proportion of them were detected regardless of the method used and the studied population.

**Table 4:** Number of common copy number variation regions between the current study and the literature

Year	Reference	Method	Samples	Breeds	CNVR	Common CNVR
2017	Rao et al. [34]	60 K Infinium II SNP BeadChip	489	4	329	229
2017	Strillacci et al. [36]	600 K Axiom® Genome-Wide Chicken Genotyping Array	96	6	564	233
2017	Gorla et al. [6]	600 K Axiom® Genome-Wide Chicken Genotyping Array	265	1	1,218	598
2017	Sohrabi et al. [35]	Whole-Genome Sequencing	24	3	5,467	986
2019	Seol et al. [37]	Whole-Genome Sequencing	60	3	609	225
2021	Fernandes et al. [15]	600 K Axiom® Genome-Wide Chicken Genotyping Array	1,461	1	5,041	1,160
2022	Chen et al. [16]	Whole-Genome Sequencing	282	6	600	149
2023	This study	600 K Axiom® Genome-Wide Chicken Genotyping Array	508	23	1,172	

### Gene annotation inside CNVR

**Fig. 4:**
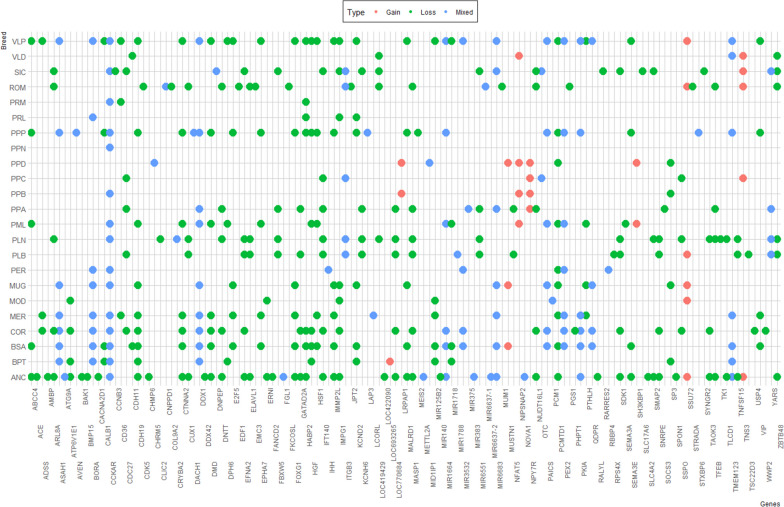
Genes distribution among the animals belonging to the investigated chicken breeds. Reported genes are the most significant as present in at least 5 animals across breeds. The colors indicate the status of copy number variation regions in which the genes were annotated (red = gain, green = loss, blue = mixed)

The dataset of the CNVR was intersected with the chicken gene database (UCSC Genome Browser losses GRCg6a). Out of the 1,172 CNVR identified in the present study, 676 (57.7%) did not incorporate any genes and 496 (42.3%) encompassed one or more genes. In detail, there were 968 genes within the genomic regions covered by the identified CNVR; 854 (88.2%) were protein-coding genes, 84 (8.7%) were miRNAs, and 30 (3.1%) were genes of as yet unknown function (LOC; Additional file 4). For the following analysis, the genes present in at least five individuals within a breed were considered, in order to evaluate those with higher incidence in the whole population. A total of 135 genes were identified and carried out to GO analyses and QTL association (Fig. 4 and Additional file 5). The Panther dataset provided the annotation information according to GO terms on the 135 selected genes. Of these genes, 75 are involved in cellular processes, 49 in biological regulations, 45 in metabolic processes, 24 in localization, 20 in multicellular organismal processes, 16 in the developmental system, 24 in signaling processes, 22 in response to stimulus, 10 in locomotion system activities, and 4 in growth processes. It is worth noting that most candidate genes were associated with production focused QTL such as body weight, breast muscle weight, fat deposition, and egg weight and quality (Additional file 5). Moreover, several CNVR were found to be conserved between breeds, both as loss and gain, acting on same genes (Fig. 4). Some of these genes that were targeted by CNVR among breeds were: *CDH19* (cadherin 19), *DACH1* (Dachshund family transcription factor 1), *IMMP2L* (inner mitochondrial membrane peptidase subunit 2), *DMD* (dystrophin), *DNPEP* (aspartyl aminopeptidase), *TMEM123* (transmembrane protein 123), *BORA* (Bora, aurora kinase A activator), *DDX1* (DEAD-box helicase 1), *IFT140* (intra-flagellar transport 140), *ARL8A* (ADP ribosylation factor like GTPase 8A), and *CCKAR* (cholecystokinin A receptor). Interestingly, the gene coding for the miRNA 6683 (*MIR6683*) was spread across the majority of the breeds (Fig. 4 and Additional file 5). All the aforementioned genes are associated with several QTL such as carcass and body weight, carcass ash and dry mater content, feed intake, skeletal development, egg quality, and average daily gain.

The GO enrichment analysis and KEGG pathways analysis invoked in DAVID yielded 126 significant enriched functional terms (48 of biological processes, 77 of cellular components, and 60 of molecular functions). In addition, 49 significant enriched pathways, including metabolic pathway, biosynthesis of aminoacyl, and the MAPK signaling pathway were detected. More details are available in the Additional file 6.

### Clustering analysis through CNVR

**Fig. 5:**
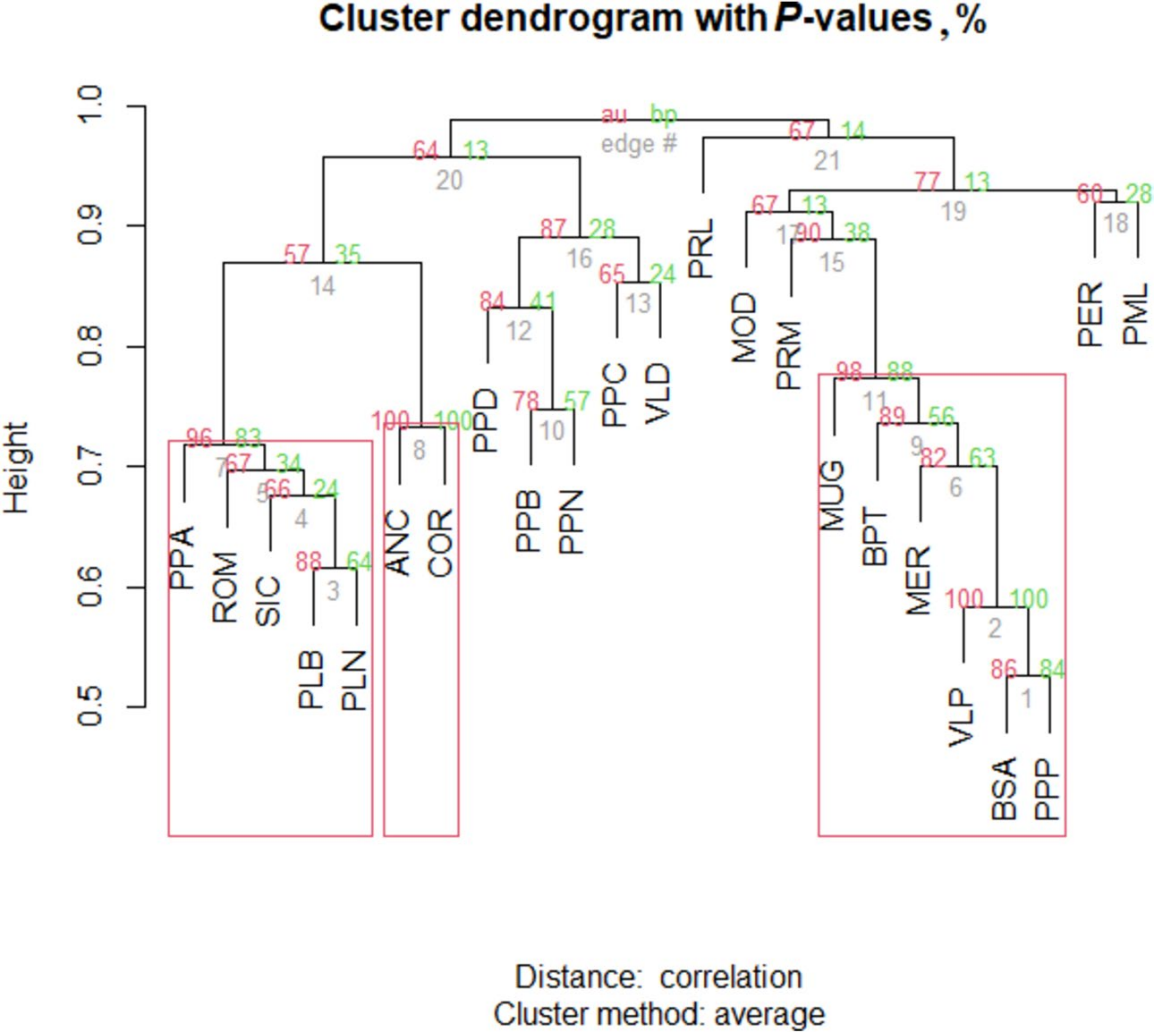
Cluster dendrogram with AU-P (approximately unbiased *P*-value) and BP-P values (bootstrap probability *P*-value) (%) among breeds. AU-P value in dark grey color, BP-P value in grey color, edge in light grey color. Breeds with high copy number variant similarities are in the red box

Figure 5 depicts the cluster-tree performed for the local Italian chicken breeds based on CNVR similarities. In the dendrogram, the branch length is not directly proportional to the genetic distance estimated among the breeds [5]. The AU-P and BP-P indicate how strongly the cluster is supported by the data and, these are reported for each node and the edge number. Here, three clusters were detected, the first composed of PPA, ROM, SIC, PLB, and PLN, the second of ANC and COR, and the third of MUG, BPT, MER, VLP, BSA, and PPP.

## Discussion

### CNV and CNVR

High-throughput, high-density genotyping technologies such as Affymetrix Axiom and/or Illumina BeadArrays, are employed in genome-wide association studies to facilitate the detection of CNV [33, 38]. For each SNP, an array platform consists of two types of hybridization probes, each specific to one of the two known alleles. The determination of the SNP genotype can be achieved by analyzing the ratios of hybridization intensities for the A and B probes (one for each allele). The CNV, such as duplications and deletions, result in an increase or decrease of the overall intensities. Additionally, in the case of large CNV spanning multiple SNP, the intensity ratios exhibit distinct patterns compared to normal disomic genomic regions. Thus, the in-silico approaches such as those described in the present study, acquire higher importance and credibility in the CNV calling [39–42].

In the present work, given the large sample size and the density of the chip used, a very large number of CNV has been reported (Table 1) compared to previous studies [6, 39, 41]. Our results are in accordance with the existing literature, which indicates that the distribution of CNV is more prominent in the first six autosomes and linked with the types 0 and 1 (Fig. 2) [39, 43, 44]. It is worth noting that most of the CNV are associated with the loss type. Additionally, the total number of animals in this population, based on the FAO DAD-ID census, is very low [36]. This is particularly noteworthy given that, overall, the inbreeding rate is high in the investigated breeds, especially for PPP [5]. However, this consideration does not strictly apply to all breeds; indeed, the SIC had a low number of CNV, despite a very high level of inbreeding [5].

The minimum length of CNV identified in COR was significantly greater than that reported in previous studies [2, 6, 36] (Table 1). The minimum length of CNV, i.e., 857 bp using the criterion of a minimum of five SNP for CNV mapping, does not coincide with the minimum length mapped by Gorla et al. [6] using the same approach.

In general, the literature reports that most CNV are of types 0 and 1 [36–38, 40], in both chicken and other species, which agrees with the present work. However, in the species *Gallus gallus*, the number of CNV is very heterogeneous between breeds [6, 36, 42], likely due to the method of analysis. In the current study, the method described by Gorla et al. [6] was used; however, those authors reported higher number of gain type CNV than loss type CNV in local Mexican chicken populations, whereas we observed more loss type CNV than gain type CNV in local Italian chicken breeds.

Strillacci et al. [36] investigated CNV and CNVR in some of the chicken breeds included in the present study, namely BPT, BSA, MER, PLB, PLN, and SIC. The number of CNV and CNVR among chromosomes for PLB and PLN in Strillacci et al. [36] was in line with those for PLB and PLN of the current study. As regards BPT, the total number of CNV and CNVR in Strillacci et al. [36] was greater than that of the current study, whereas in BSA and MER the total number of CNV and CNVR was lower. The animals used in our study were more recent and it is likely they have undergone new recombination events since the earlier studies. There are multiple genomic phenomena that can cause changes in the distribution of CNV within the genome over time.

Approximately 40% of the CNVR (480 out of 1,172) detected in our study are conserved and the remaining 692 are new and represent single regions. As reported in Table 3, the proportion of CNVR in common between the present work and past studies is moderate to high. Moreover, regardless of the breeds included in other studies, the CNVR detection is mainly influenced by sample size and the algorithm, and the technology used to map CNV (i.e., high density SNP array, low density SNP array or whole genome sequencing). However, a strength of the present study is the use of intensity signals from genotyping that allowed strong validation of the results [45, 46].

As expected from previous studies, chromosome 16 stood out with a high proportion of its length covered by CNVR (36.5%; Fig. 3 and Table 3), which can be attributed to two main factors. Firstly, chromosome 16 is the shortest autosome of the genome of *Gallus gallus*. Secondly, it harbors the MHC, which is known to be subject to genomic CNV [43]. This complex contains a cluster of genes responsible for the encoding of the proteins present on cell surfaces, aiding the immune system in identifying exogenous substances. Many of these genes contribute to immune responses with specific alleles at some loci that potentially have a major role in the genetic mechanisms of resistance to infectious diseases [43, 46]. The presence of CNV on chromosome 16 could be crucial in conferring or not a particular resistance to diseases; on the other hand, the presence of CNV as deletion could contribute to susceptibility to the diseases. In the present study, only the ANC breed had several CNV on chromosome 16. However, the chromosome coverage percentage identified was very low in this study when compared with previous studies [20, 44]. Chromosome 16 is very interesting as it contains the key genes for resistance to infectious diseases and therefore it is subject to various natural genetic modifications due to the presence of a large number of polymorphic sites (i.e., avian influenza, Rous sarcoma disease, avian leucosis, *Escherichia coli*, *Salmonella enteritidis*) [20, 44].

The CNV identified in the VLD and PPP breeds are primarily present on the first seven autosomes, while the remaining breeds showed a more evenly pattern of CNV coverage throughout the genome. The distribution on the first seven autosomes was expected as they are significantly larger than the others. Notably, the ANC and COR breeds exhibited a well-balanced distribution of CNV across all chromosomes (Additional file 2). In addition, the physical distribution of CNVR on chromosomes is in accordance with Gorla et al. [6].

Out of the identified CNVR, 58.9% were observed in only one individual, 20.1% in two individuals, 6.1% in three individuals, 4.8% in four individuals, and 10.1% in more than five individuals. The high proportion of the singleton has been previously reported by Yi et al. [20] (68.8%), Strillacci et al. [36] (75%), and Han et al. [47] (76.5%). This finding confirms the existence of segregating CNV among individuals, as highlighted by the large proportion of singleton CNVR.

### CNVR annotation and QTL

Changes in the CNV can cause the deletion or duplication of genes, and these changes can alter gene expression [48]. Therefore, identifying these affected genes is an important part of studies on CNV and CNVR. Some of the annotated genes within the CNVR have been already associated to functional traits in chickens (Additional file 5). The CNVR located on chromosome 1, shared amongst different breeds, were used to annotate several genes, the most noteworthy being *CACNA2D1* (calcium voltage-gated channel auxiliary subunit alpha 2 delta 1) which is related to muscle contraction [49]; *DMD* (dystrophin), one of the most important factors for muscle development and structural stability of the tissue [50]; and *DACH1*, involved in skeletal development and inhibitor of growth factor beta [51, 52]. The CNVR that contains the latter gene was identified in the PPP breed, which is a small size breed [53]. Some other genes were *BORA,* related to cell growth and divisions and consequently influences on whole growth traits [54]; *IMMP2L*, involved in the reproduction traits and fertility [55]; and *TMEM123*, associated with adipogenic differentiation of chicken preadipocytes [56].

The CNVR on chromosome 3 harbor several genes, however, the most relevant is *DDX1* that strengthens the immunity response and therefore it may have played a role in the acquired resistance of local breeds to environmental stimuli [57]. On chromosome 4, *CCKAR* is important for body weight and its variants have a central role in the diversification of gene expression [58]. The genes *IFT140* and *ARL8A* were identified on chromosomes 14 and 26, respectively; these genes are associated with eggs and fertility. In detail, *IFT140* is involved in the maturation and efficiency of seminal cells and *ARL8A* in both egg production and brown pigmentation [59–61]. These findings are important due to the low efficiency of these local breeds in terms of fertility and egg production [62].

Several genes are of particular interest due to their presence across breeds and they include *SLC4A2* (solute carrier family 4 member 2) on chromosome 2, *CCNB3* (cyclin B3) on chromosome 4, and *DNPEP* (aspartyl aminopeptidase) on chromosome 7. These genes have been linked to muscle development and tissue-specific biological processes in muscle [63]. Noteworthy, the gene encoding the miRNA *MIR6683* is present in CNVR 621 which has been identified in the BSA, MER, MUG, PPA, SIC and VLP breeds, and is associated with sex determination (Additional file 5) [64].

Another useful information obtained in this study through KEGG analysis is the MAPK signaling pathway, which plays an important role in complex cellular programs like proliferation, differentiation, development, transformation, and apoptosis [65]. These cellular events are critical to immune development and some other processes. Importantly, mutations that constitutively activate or fail to regulate the MAPK signaling properly cause inflammatory disease, including several chicken diseases [66, 67].

Additionally, QTL from the chicken QTLdb were downloaded to verify the overlap with the identified CNVR. Since the confidence interval of some QTL was very wide, only those shorter than 5 Mb and involving loci with high production impact were considered. Out of the 135 gene-containing regions, 39 overlapped with 1,475 QTL, mainly including QTL for body weight, body size, carcass characteristics, egg production, characteristics of egg yolk and albumen, fat deposition, and feed intake (Additional file 5).

### Clustering analysis

Some breeds created precise clusters based on the characterization of the CNVR and this contributed to the definition of the classes of the breeds under investigation. Several papers reported procedures to define classes on the basis of CNVR [36, 41]; however, as the length of the arms within the dendrogram are not directly proportional to the estimated genetic distance between the samples, this classification is difficult. Based only on AU-P, the cluster (edge) with AU-P > 95% is the most plausible method [32]. Edge is the order in which the clusters are built: more closely related samples have smaller edge number, whereas higher edge number reflects clusters formed later in the evolutionary process of the breed [36]. The dendrogram in Fig. 5 was carried out by breed and not by animal due to the complexity of the interpretation. Our results do not properly represent the distribution of the breeds and the separation among breeds, due to the limit that the CNV locus must contain at least 5 SNP probes in the statistical analysis. The exclusion of some breed-specific CNV (with less than 5 SNP) could have affected the clustering [36].

## Conclusion

Genetic variability and diversity within and between 23 local Italian chicken breeds using CNV markers were assessed. The CNV analysis has not effectively distinguished breeds based on their breeding history and genetic identity. The findings lay the groundwork for acknowledging the local Italian chicken population as a vital repository of genetic diversity, using high-density SNP genotypes. The study permitted the development of a CNV map in local populations well adapted to harsh environments. Interestingly, some of the CNV are located in the chromosomal regions where crucial functional genes have been annotated, such as the MHC region on chromosome 16. In conclusion, this study confirmed the presence of genetic and genomic variability in local Italian chicken breeds and supports the opportunity to utilize them for conservation purposes.

### Supplementary Information

**Additional file 1.** Number of CNV called using different algorithms.

**Additional file 2. **Number of CNVR detected per chromosome; each color represents a single breed.

**Additional file 3.** Distribution of CNVR lengths identified with PennCNV.

**Additional file 4. **List of CNVR and respective annotated genes.

**Additional file 5. **Candidate genes identified in at least 5 animals among breeds.

**Additional file 6.** Detailed list of the annotated genes investigated through DAVID.

## Data Availability

The datasets generated and/or analyzed during the current study are not publicly available. They can be made available from the corresponding author upon reasonable request.

## Abbreviations

ANC: Ancona
ARL8A: ADP ribosylation factor like GTPase 8A
BAF: B allele frequency
BORA: Bora, aurora kinase A activator
BPT: Bionda Piemontese
BSA: Bianca di Saluzzo
CACNA2D1: Calcium voltage-gated channel auxiliary subunit alpha 2 delta 1
CCKAR: Cholecystokinin A receptor
CCNB3: Cyclin B3
CDH19: Cadherin 19
CNV: Copy number variant
CNVR: Copy number variation region
COR: Cornuta di Caltanissetta
DACH1: Dachshund family transcription factor 1
DAVID: The database for annotation visualization and integrated discovery
DDX1: DEAD-box helicase 1
DMD: Dystrophin
DNPEP: Aspartyl aminopeptidase
GO: Gene Onthology
IFT140: Intra-flagellar transport 140
IMMP2L: Inner mitochondrial membrane peptidase subunit 2
KEGG: Kyoto Encyclopaedia of Genes and Genomes
LRR: Log R ratio
MER: Mericanel della Brianza
MOD: Modenese
MUG: Mugellese
PER: Ermellinata di Rovigo
PLB: Livorno Bianca
PLN: Livorno Nera
PML: Millefiori di Lonigo
PPA: Padovana Argentata
PPB: Polverara Bianca
PPC: Padovana Camosciata
PPD: Padovana Dorata
PPN: Polverara Nera
PPP: Pepoi
PRL: Robusta Lionata
PRM: Robusta Maculata
ROM: Romagnola
QTL: Quantitative trait loci
SIC: Siciliana
SLC4A2: Solute carrier family 4 member 2
SNP: Single nucleotide polymorphisms
TMEM123: Transmembrane protein 123
VLD: Valdarnese
VLP: Valplatani
