# Supplementary figures and images for: Genome-wide investigation to assess copy number variants in the Italian local chicken population
Source: J Anim Sci Biotechnol. 2024 Jan 3;15:2. doi: 10.1186/s40104-023-00965-7 (PMC10763469; doi:10.1186/s40104-023-00965-7)

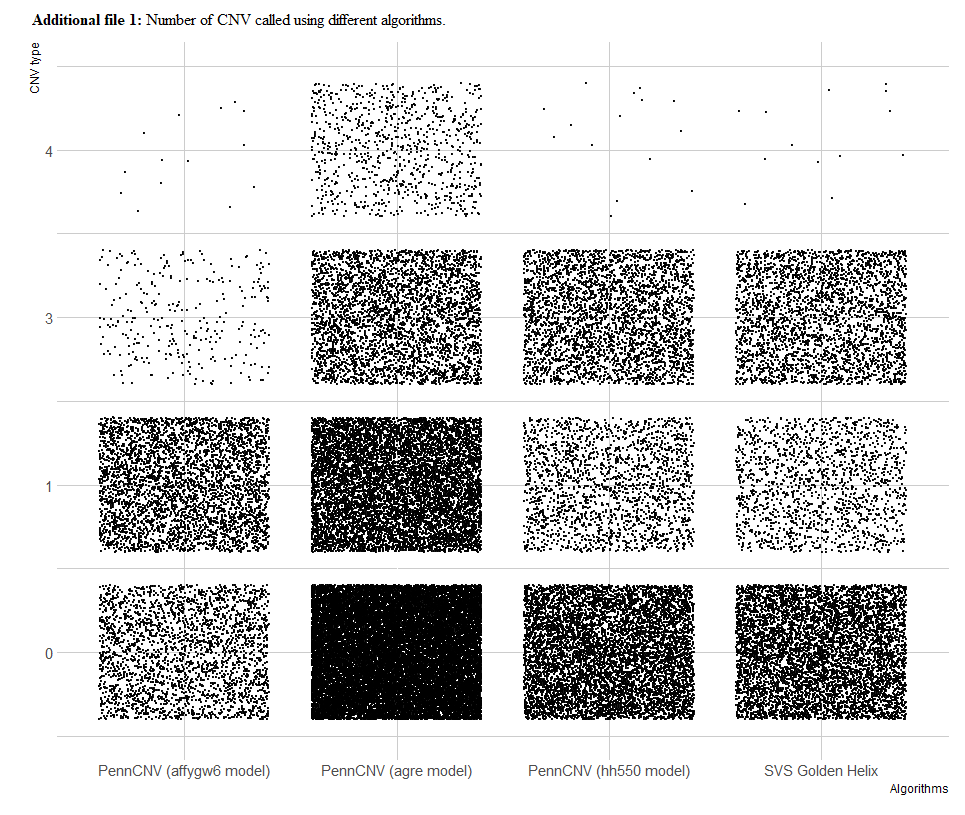

Supplement: Supplementary file 1 — Additional file 1. Number of CNV called using different algorithms. [file 40104_2023_965_MOESM1_ESM.png]

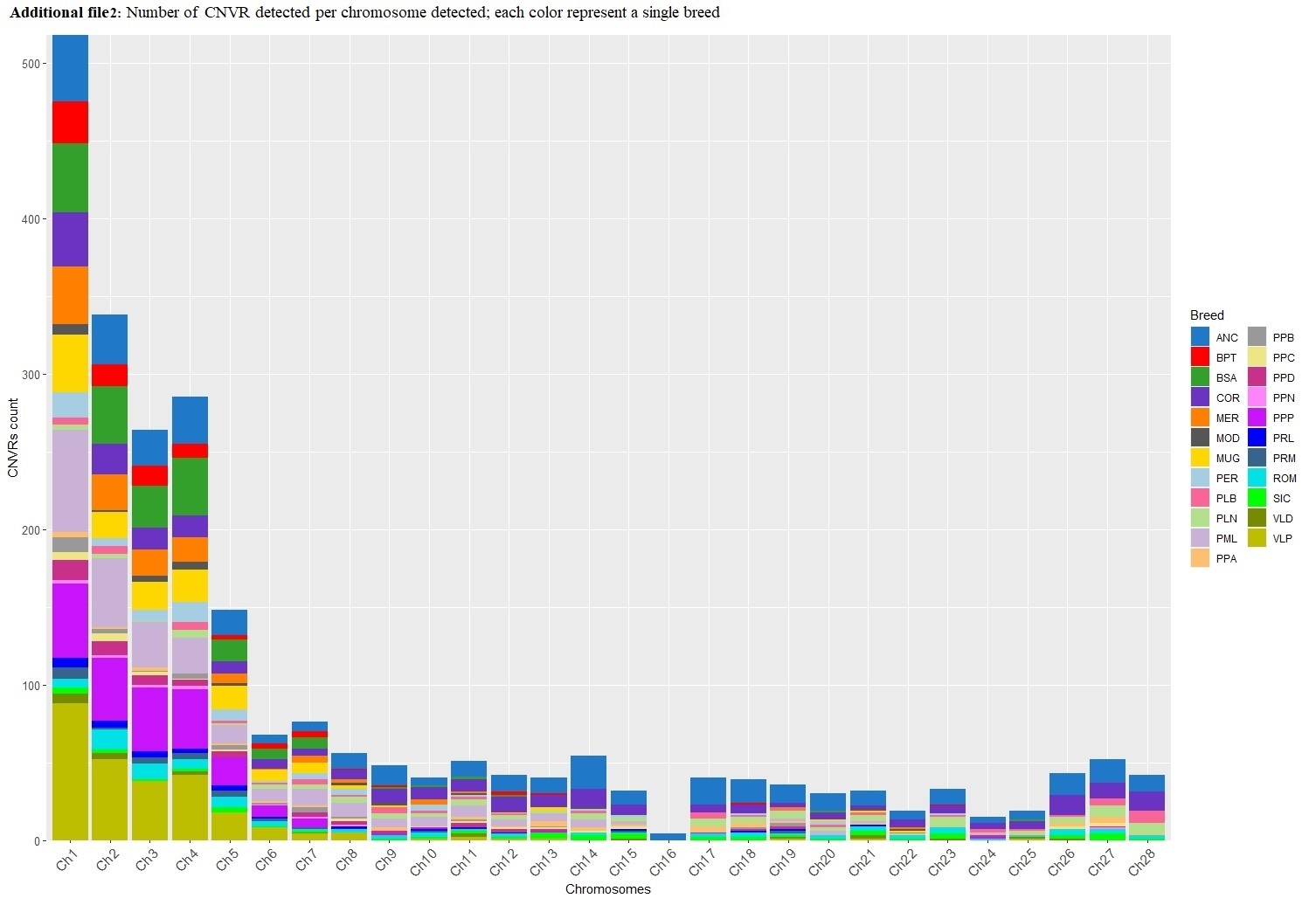

Supplement: Supplementary file 2 — Additional file 2. Number of CNVR detected per chromosome; each color represents a single breed. [file 40104_2023_965_MOESM2_ESM.jpeg]

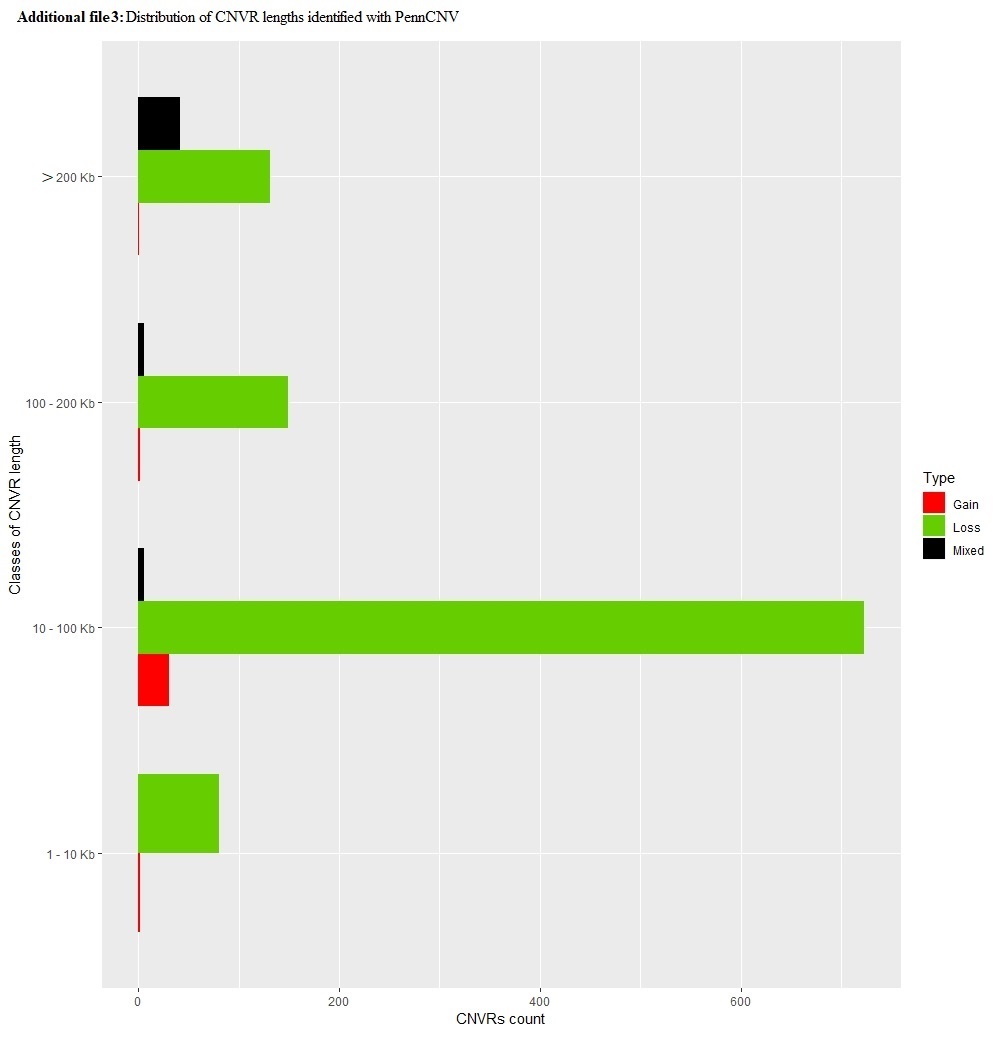

Supplement: Supplementary file 3 — Additional file 3. Distribution of CNVR lengths identified with PennCNV. [file 40104_2023_965_MOESM3_ESM.jpeg]
